# Popliteal Plexus Block as a Potential Alternative to IPACK Block in Total-Knee Arthroplasty: Anatomical Rationale, Clinical Evidence, and Ergonomic Advantages

**DOI:** 10.3390/jcm15155818

**Published:** 2026-07-25

**Authors:** Sung Jun Kim, Siwook Chung, Jae Hoo Park, Hye Joo Yun

**Affiliations:** 1 Department of Anesthesiology and Pain Medicine, Eunpyeong St. Mary’s Hospital, College of Medicine, The Catholic University of Korea, Seoul 03312, Republic of Korea; 2 Department of Anesthesiology and Pain Medicine, Incheon St. Mary’s Hospital, College of Medicine, The Catholic University of Korea, Incheon 21431, Republic of Korea

**Keywords:** total-knee arthroplasty, popliteal plexus block, IPACK block, motor-sparing regional anesthesia, enhanced recovery after surgery

## Abstract

Total-knee arthroplasty (TKA) is associated with substantial acute postoperative pain. While the adductor canal block (ACB) is widely used to provide motor-sparing anterior knee analgesia, effective management of posterior knee pain remains an important clinical challenge. The infiltration between the popliteal artery and the capsule of the posterior knee (IPACK) block was developed to address posterior capsular pain but may be associated with technical and ergonomic limitations. More recently, the popliteal plexus block (PPB) has emerged as a novel motor-sparing alternative. This narrative review summarizes the anatomical basis, current clinical evidence, and practical considerations of combining the ACB with the PPB, with particular emphasis on its potential role as an alternative to the IPACK block within Enhanced Recovery After Surgery (ERAS) pathways. Current anatomical and early clinical evidence suggests that the PPB selectively targets the terminal articular branches around the adductor hiatus while preserving the major motor nerve trunks. Available randomized clinical trials indicate that the PPB provides analgesia comparable to IPACK while maintaining motor function, with additional practical advantages including shorter procedural time and, in individual studies, reduced postoperative opioid consumption and postoperative nausea. Overall, the combined ACB and PPB approach appears to be a promising motor-sparing strategy for perioperative analgesia after TKA. However, the current evidence is derived predominantly from cadaveric studies, early-phase randomized trials, and single-center clinical investigations. Therefore, larger multicenter studies with longer follow-ups are needed to establish its long-term efficacy, safety, and role relative to the established IPACK technique.

## 1. Introduction

Total-knee arthroplasty (TKA) is widely recognized as one of the most successful orthopedic procedures, offering substantial pain relief, restoration of joint function, and significant improvements in quality of life for patients with severe osteoarthritis, rheumatoid arthritis, and other degenerative joint diseases [1]. Driven by an aging population and increasing obesity rates, the global demand for TKA is rising exponentially. In the United States alone, the annual procedure volume is projected to exceed 3.48 million by 2030, with further substantial growth anticipated by 2050 [2].

This surge imposes a profound economic burden on healthcare systems, fostering strong clinical and policy-driven initiatives to reduce the length of hospital stay (LOS) and transition toward outpatient arthroplasty. Notably, the removal of TKA from the inpatient-only list by the U.S. Centers for Medicare & Medicaid Services (CMS) in 2018 institutionally accelerated this paradigm shift toward ambulatory surgery [3,4].

The extensive bone resection, capsular retraction, and substantial soft tissue injury render acute postoperative pain following TKA among the most severe of all orthopedic procedures [5]. Effective initial pain management extends beyond merely improving patient comfort; it is a pivotal determinant of success in Enhanced Recovery After Surgery (ERAS) protocols. Optimal analgesia facilitates early ambulation, the restoration of active range of motion (ROM), shorter LOS, and the prevention of severe postoperative complications such as deep vein thrombosis (DVT) and pulmonary embolism [6]. Recent studies indicate that despite the implementation of ERAS protocols and advanced regional analgesia, nearly 30% of patients still experience moderate-to-severe early postoperative pain, highlighting the critical consequences of inadequate initial analgesic interventions [3,7].

Historically, postoperative pain management has relied primarily on intravenous patient-controlled analgesia (IV-PCA) with opioids. However, concerns regarding opioid-related systemic adverse effects—including postoperative nausea and vomiting (PONV), respiratory depression, urinary retention, ileus, and delirium— have driven efforts to reduce opioid use, as these complications may delay rehabilitation and prolong hospital stay [8]. Furthermore, in the context of the ongoing global opioid epidemic, increasing emphasis has been placed on opioid-sparing or opioid-free analgesic protocols to minimize perioperative opioid consumption and optimize postoperative recovery following orthopedic surgery [9,10].

Against this backdrop, the contemporary analgesic paradigm has definitively shifted toward multimodal analgesia pathways centered on ultrasound-guided regional anesthesia. Regional anesthetic techniques have also undergone continuous evolution [3,9,10]. Although traditionally providing potent analgesia, the femoral nerve block (FNB) and sciatic nerve block (SNB) induce severe motor weakness in the quadriceps and lower leg muscles, thereby precipitating their phase-out from modern ERAS pathways [11,12,13]. The historical evolution and clinical limitations of these regional anesthesia paradigms are summarized in Table 1.

While the adductor canal block (ACB), which preserves quadriceps motor function while providing sensory analgesia to the anterior knee, has become a widely accepted component of contemporary regional anesthesia for TKA [19], its inherent inability to control posterior knee pain creates a significant clinical “blind spot” wherein 67% to 89% of patients report severe posterior pain postoperatively [15]. To address this severe posterior pain without inducing motor deficits such as foot drop [5], the infiltration between the popliteal artery and the capsule of the knee (IPACK) block was developed and has since been widely adopted [16,20].

Building upon this anatomical foundation, the popliteal plexus block (PPB) has recently emerged as a motor-sparing regional anesthesia technique that may address some of the technical and ergonomic limitations associated with the IPACK block [15,17]. This narrative review aims to summarize the current anatomical, technical, and clinical evidence regarding the role of the PPB in postoperative pain management following TKA. Specifically, the review first outlines the methodological framework and the anatomical basis of both techniques. It then examines the available clinical evidence, including analgesic efficacy, procedural characteristics, and ergonomic considerations, while also discussing the current limitations of the evidence base. Finally, the review highlights existing knowledge gaps and future research directions regarding the role of the PPB in contemporary TKA pathways.

Because the PPB remains an emerging technique with a limited number of clinical studies, we intentionally chose a narrative review design to integrate anatomical concepts, mechanistic insights, and the available clinical evidence rather than to perform a formal systematic review.

### Literature Search Strategy

To ensure a reproducible and balanced review, we performed a structured literature search across PubMed/MEDLINE, Embase, and Cochrane CENTRAL for studies published between January 2012 and May 2026. The search strategy included the following query: ((“Total Knee Arthroplasty” OR “TKA”) AND (“Popliteal Plexus Block” OR “PPB” OR “IPACK block” OR “Adductor Canal Block” OR “ACB”) AND (“Analgesia” OR “Motor-sparing” OR “Anatomy” OR “ERAS” OR “Ergonomic”)). Two independent reviewers screened 531 records. Following title and abstract screening, 471 records were excluded (e.g., non-TKA procedures, non-human studies, or studies not involving peripheral nerve blocks). The remaining 60 articles underwent full-text review, of which 31 were excluded because of substantial methodological confounding or insufficient reporting of quantitative clinical outcomes, resulting in 29 comparative clinical studies for the primary evidence synthesis. Because systematic database searches may under-represent detailed anatomical studies, a targeted manual search of specialty regional anesthesia journals and reference lists was additionally performed, identifying 26 foundational publications. Overall, 55 studies were included in this review. Because this review was designed as a narrative review intended to integrate anatomical concepts, mechanistic insights, and emerging clinical evidence rather than quantitatively synthesize comparative effectiveness, a formal risk-of-bias assessment using standardized tools (e.g., RoB 2 or ROBINS-I) was not performed. The included clinical studies were critically appraised for potential sources of bias, including small sample sizes, single-center designs, and operator dependence. This search strategy was designed to evaluate the current evidence regarding the PPB as a potential motor-sparing alternative to the iPACK block, rather than as its definitive replacement, thereby providing a balanced interpretation of the available literature.

## 2. The Complex Anatomical Innervation Mechanisms of the Knee Joint

A comprehensive understanding of the three-dimensional innervation of the knee joint is fundamental to developing an effective regional anesthesia strategy. Rather than relying solely on dermatomal distribution, the complex sensory innervation of the knee should be considered in terms of its dermatomal, myotomal, osteotomal, and capsular components [21]. Although regional anesthesia is often described according to dermatomal coverage, this approach may not fully explain postoperative pain following deep orthopedic procedures such as TKA, where pain arises from extensive osteotomies, capsular manipulation, ligament resection, and implant insertion rather than from the skin alone [3,22].

### 2.1. Concepts of Dermatome, Myotome, Osteotome, and Hilton’s Law

The sensory innervation of the knee reflects its complex embryological development and can be broadly categorized into dermatomal, myotomal, osteotomal, and capsular components [23,24]. While dermatomes describe the cutaneous sensory distribution, osteotomal and capsular innervation are primarily responsible for transmitting pain arising from deep musculoskeletal structures.

**Figure 1:**
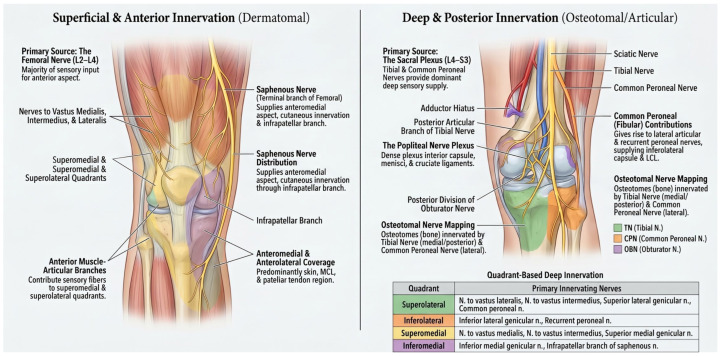
Dermatomal vs. Osteotomal and capsular innervation in the knee joint. Illustration created using Gemini (3.5 Flash) based on the anatomical concepts described by Tran et al. (2018, 2019) [22,29,30], Burckett-St Laurant et al. (2016) [31], Bendtsen et al. (2016) [27], and Roberts et al. (2020) [32].

During TKA, the surgical incision traverses the anteromedial dermatomes and activates the cutaneous branches of the femoral nerve (L2–L4), resulting in superficial incisional pain [25]. However, the characteristic postoperative pain following TKA is predominantly generated by extensive bone resection, ligament release, capsular manipulation, and soft-tissue traction, which stimulate nociceptors within the osteotomal and capsular structures rather than the skin alone. The anatomical relationship between dermatomal and osteotomal innervation is illustrated in Figure 1 [26,27].

The complexity of articular innervation can be understood in the context of Hilton’s Law, proposed by the nineteenth-century anatomist John Hilton. This principle states that the nerve supplying a muscle crossing a joint also provides sensory branches to the joint capsule and the overlying skin [28]. Because knee movement depends on the coordinated function of the quadriceps, hamstrings, gastrocnemius, popliteus, and other periarticular muscles, the corresponding nerves arising from the lumbar and sacral plexuses also contribute articular branches that participate in knee joint innervation [22,26,27].

### 2.2. Anterior Innervation: Anterior Capsule and Osseous Structures

The anterior and medial compartments of the knee joint are primarily innervated by the anterior divisions of the femoral nerve (L1–L4) and the obturator nerve (L2–L4), both originating from the lumbar plexus [21]. As the femoral nerve descends through the thigh, it divides into robust motor and sensory branches. The intense innervation of the anterior osseous structures and capsule by the deep articular branches of the femoral nerve—specifically the nerves to the vastus medialis (NVM), lateralis, and intermedius [29]—is structurally complemented by the saphenous nerve, which traverses the adductor canal to provide infrapatellar sensory coverage to the anteromedial knee and overlying tissue [33]. Given the absolute dominance of the femoral nerve system over the anterior compartment, the simultaneous blockade of the NVM and the saphenous nerve within the adductor canal constitutes the physiological basis for the exceptional efficacy of the ACB in controlling anterior TKA pain [12,15,29,31].

### 2.3. Posterior Capsular Innervation: The Complexity of the Popliteal Plexus

The posterior capsule of the knee, the epicenter of recalcitrant pain following TKA, possesses a substantially more multidimensional anatomical innervation [34]. This complexity arises from the convergence of the sciatic nerve (L4-S3) from the sacral plexus and the posterior division of the obturator nerve (PON) from the lumbar plexus, which interlace to form the dense, reticular ‘popliteal plexus’ [14,22,35].

Recent meticulous cadaveric dissections by Tran et al. and other anatomists have precisely mapped the origins, trajectories, and frequencies of the articular branches targeting the posterior capsule [15,22].

#### 2.3.1. Tibial Nerve

As the medial division of the sciatic nerve, the tibial nerve descends vertically through the midline of the popliteal fossa, projecting the highest volume of articular branches broadly across the posterior capsule. Cadaveric studies consistently demonstrate tibial nerve articular branches distributing throughout the posterior capsule in 100% of specimens [22,35,36].

#### 2.3.2. Posterior Division of the Obturator Nerve (PON)

Descending along the medial thigh anterior to the adductor magnus, the PON traverses the adductor hiatus alongside the femoral vessels to enter the popliteal fossa. It subsequently travels in close apposition to the anteromedial aspect of the popliteal artery, providing dense sensory innervation predominantly to the superomedial quadrant of the posterior capsule. This territory significantly overlaps with that of the tibial nerve [14,22,27].

#### 2.3.3. Common Fibular (Peroneal) Nerve (CFN) and the Main Sciatic Nerve Trunk

Branches derived from the CFN (the lateral division of the sciatic nerve) and occasionally from the main sciatic trunk innervate the superolateral aspect of the posterior capsule. Cadaveric mappings identify CFN and main sciatic branches in approximately 53% and 20% of specimens, respectively [22,32].

#### 2.3.4. Formation of the Popliteal Plexus

**Figure 2:**
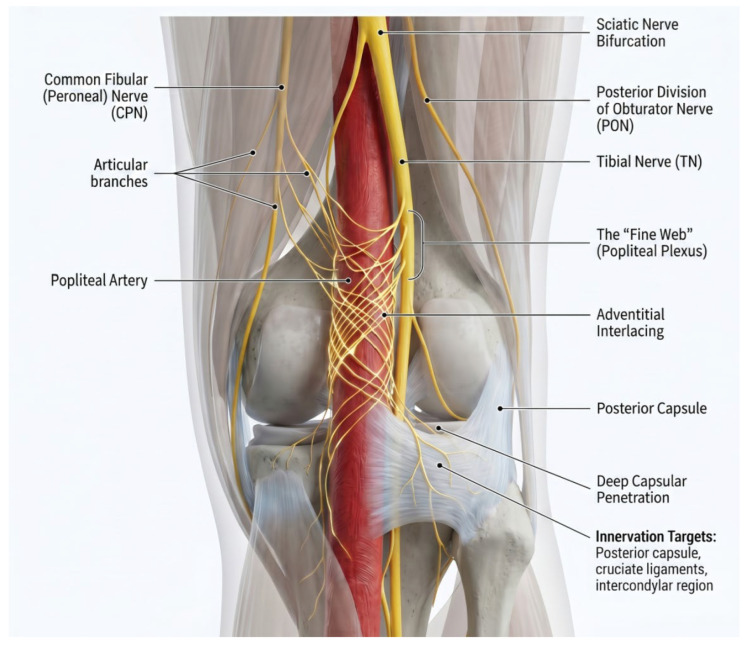
Three-dimensional anatomical structure of the popliteal plexus: innervation of the posterior knee. Illustration created using Gemini (3.5 Flash) based on the anatomical concepts described by Tran et al. (2019) [22,30], Bendtsen et al. (2016) [27], Runge et al. (2017) [18], Roberts et al. (2020) [32], and Fujino et al. (2025) [17].

The terminal articular filaments derived from the tibial, common fibular, and posterior obturator nerves intertwine around the adventitia of the popliteal vessels to form the reticular ‘popliteal plexus.’ These fine neural filaments course distally along the inferomedial aspect of the lateral femoral condyle, deeply penetrating the posterior capsule to transmit nociceptive signals, as structurally mapped in Figure 2 [15,22,35,36].

#### 2.3.5. Clinical Implications of Popliteal Plexus Anatomy for Posterior Knee Analgesia

The paramount clinical insight derived from these anatomical data is that posterior knee pain following TKA cannot be simplistically dismissed as mere “sciatic nerve pain.” The persistence of severe superomedial capsular pain following an isolated sciatic nerve block when the lumbar plexus-derived PON is spared underscores a critical clinical reality: precisely targeting the popliteal plexus itself, as the ultimate convergence of these disparate neural pathways, represents the most anatomically logical and physiologically sound approach to comprehensive posterior analgesia [15,17,22,34,35].

## 3. Complementary Mechanisms of Combined ACB and PPB

The cardinal challenge in modern regional anesthesia is resolving the physiological dilemma: achieving complete nociceptive blockade while rigorously sparing the motor nerve function essential for ambulation, weight-bearing, and joint mobilization [3,11,37]. Based on the intricate anatomical innervation maps discussed previously, the concurrent application of an ACB for the anterior thigh and a PPB for the posterior popliteal region currently represents the most optimal therapeutic strategy [15,38].

### 3.1. Clinical Role and Anatomical Limitations of the ACB

While the traditional FNB, administered just inferior to the inguinal ligament, guarantees excellent anterior analgesia, it invariably induces complete quadriceps paralysis that frequently results in knee buckling and postoperative falls [12,26,39]. This profound motor deficit directly conflicts with contemporary ERAS protocols, which mandate active ambulation on postoperative day zero [10,11,13,40].

To circumvent this, the ACB was developed to target the fascial corridor deep to the sartorius muscle in the middle third of the thigh [12,19]. The adductor canal primarily houses the saphenous nerve (a purely sensory nerve) and the nerve to the vastus medialis (NVM), which provides motor supply to the vastus medialis but critically contributes sensory branches to the anteromedial capsule [3,31]. Ultrasound-guided local anesthetic deposition within this space preserves over 90% of quadriceps motor function (notably the rectus femoris and vastus lateralis) while effectively mitigating sharp anteromedial knee pain [41,42]. Anatomically constrained as a fascial plane block within the anterior thigh [3,15], the ACB rarely facilitates local anesthetic transit into the posterior popliteal fossa. Although high-volume injections occasionally track distally through the adductor hiatus to reach posterior neural structures, this spread remains notoriously inconsistent and clinically irreproducible [26,43], leaving patients with an isolated ACB vulnerable to severe, burning posterior knee pain in the post-anesthesia care unit (PACU) and precipitating a vicious cycle of heavy intravenous opioid rescue [15,44].

### 3.2. Anatomical Basis and Clinical Evidence for the Motor-Sparing Profile of the PPB

Developed to address the limitations of traditional proximal sciatic nerve blocks—such as the potential for motor blockade, including foot drop, reduced plantarflexion, and difficulty in postoperative neurological assessment of the CFN—the PPB adopts a distal perivascular targeting approach [5,15,34]. Under ultrasound guidance, the superficial femoral artery is traced distally from the apex of the femoral triangle. As the artery passes through the adductor hiatus and becomes the popliteal artery, the target injection plane is identified [15,34].

The needle is then advanced to the fascial plane between the femoral shaft and the posterior wall of the popliteal artery (typically using a lateral-to-medial approach, with the needle tip positioned at approximately the 1–2 o’clock or 10 o’clock position relative to the artery, depending on the specific approach) [15]. Local anesthetic deposited at this site is intended to spread within the perivascular fascial plane of the popliteal fossa, thereby targeting the reticular popliteal plexus, which is formed by contributions from the posterior division of the obturator nerve and the articular branches of the tibial and common fibular nerves [35]. Because this injection site is anatomically separate from the main trunks of the sciatic and tibial nerves, plantarflexion and dorsiflexion of the foot are generally preserved in the available clinical studies [22]. Accordingly, this approach is designed to provide a selective sensory blockade by preferentially targeting the terminal sensory branches supplying the posterior capsule of the knee [5,15,17].

When compared with a selective tibial nerve block (TNB), the PPB demonstrated non-inferiority in achieving early postoperative rehabilitation goals while preserving significantly greater distal foot motor strength at 6 h after the nerve block. Specifically, ankle dorsiflexion was better preserved in the PPB group than in the TNB group (87.7% ± 11.4% vs. 74.0% ± 16.5%, *p* < 0.001), as was ankle plantarflexion (90.9% ± 10.3% vs. 72.1% ± 16.0%, *p* < 0.001). However, these differences were no longer statistically significant at 24 h postoperatively [45].

### 3.3. Combined Anterior and Posterior Knee Analgesia (ACB + PPB)

The combination of ACB (or femoral triangle block [FTB]) and PPB is intended to provide complementary analgesic coverage of the anterior and posterior aspects of the knee while preserving motor function [15,46].

#### 3.3.1. Reduction in Early Opioid Consumption

A recent randomized controlled trial (RCT) by Stebler et al. demonstrated that the addition of the PPB to a continuous FNB reduced morphine-equivalent consumption during the first 12 h postoperatively (6.1 mg vs. 10.0 mg; *p* = 0.04) [15]. Although this difference was not sustained over time, these findings suggest a potential opioid-sparing effect during the early postoperative period [15,41].

#### 3.3.2. Decreased Incidence of Posterior Pain in the PACU

In the same study, the incidence of lateroposterior knee pain in the PACU was lower in the PPB group than in the control group (34.4% vs. 61.8%, *p* = 0.03) [15]. These findings suggest that the PPB may provide more effective coverage of the posterior knee sensory innervation, consistent with its proposed anatomical rationale [15,41,47].

#### 3.3.3. Facilitation of Early Rehabilitation

Comparative studies evaluating the combined adductor canal block and popliteal plexus block (APB) versus local infiltration analgesia (LIA) have reported lower resting and dynamic visual analog scale (VAS) pain scores, as well as greater ambulation distances on postoperative day 2 in the APB group [38]. In the available studies, no ankle muscle weakness was observed, suggesting preservation of distal motor function and potential compatibility with ERAS protocols [15,17,48]. In addition, a recent double-blind prospective trial reported that patients receiving the PPB combined with an adductor canal block achieved early rehabilitation milestones, such as standing, walking, and exercising, sooner than those receiving anterior blocks alone (*p* < 0.05), without reported falls or clinically significant motor weakness [49].

#### 3.3.4. Complementary Analgesic Mechanisms of Combined ACB and PPB

Taken together, the available evidence suggests that the combination of the ACB and PPB may provide complementary analgesic coverage of the anterior and posterior aspects of the knee while preserving motor function [15,38,50]. By targeting the anterior compartment through the adductor canal and the posterior compartment through the popliteal plexus, this combined approach has the potential to optimize postoperative analgesia and facilitate early rehabilitation.

## 4. In-Depth Comparative Analysis: PPB Versus IPACK Block

As the importance of managing posterior knee pain after total-knee arthroplasty has become increasingly recognized, the IPACK block has been widely adopted in clinical practice over the past several years [15,16]. Although the IPACK block has demonstrated effective postoperative analgesia, it may be associated with technical and ergonomic challenges depending on the procedural approach and patient characteristics [17,46]. With the recent introduction of the PPB, comparative evaluation of these two techniques has become increasingly relevant for informing contemporary clinical practice. Based on the available cadaveric studies and early clinical trials, this section compares the two techniques with respect to their analgesic efficacy, procedural characteristics, and ergonomic considerations.

### 4.1. Clinical Efficacy and Analgesic Profiles

A recent randomized non-inferiority trial comparing the PPB with the IPACK block, in which all patients received a baseline FTB, demonstrated non-inferior analgesic efficacy between the two techniques [17]. These findings suggest that the PPB may represent a promising motor-sparing alternative to the IPACK block. In the same study, patients receiving a PPB exhibited lower postoperative fentanyl consumption and a reduced incidence of postoperative nausea and vomiting (PONV) compared with the IPACK group [17]. Although these findings suggest a potential opioid-sparing benefit that may facilitate postoperative recovery within ERAS pathways [3,40,51], they are currently derived from a limited number of clinical studies, and their broader clinical significance should be confirmed in future multicenter randomized trials.

### 4.2. Sonoanatomy and Procedural Differences

Although both IPACK and the PPB share the objective of anesthetizing the posterior articular branches, they utilize fundamentally different sonographic levels and target distinct fascial spaces [15,50].

#### 4.2.1. The IPACK Approach

The target of the IPACK block is the narrow interspace between the anterior wall of the popliteal artery and the posterior aspect of the femoral shaft, immediately adjacent to the posterior knee capsule. In the original (distal) IPACK technique, the ultrasound transducer is positioned transversely over the popliteal fossa at the level of the superior border of the femoral condyles to identify the popliteal artery and the adjacent femoral cortex. The needle is then advanced in-plane from medial to lateral into the space between the popliteal artery and the posterior capsule, where local anesthetic is deposited to block the terminal articular branches innervating the posterior knee capsule while preserving motor function [30]. Subsequently, a modified proximal IPACK approach was introduced, in which the transducer is placed on the medial aspect of the distal thigh approximately 1–2 fingerbreadths proximal to the patella. After identifying the femoral shaft and the pulsatile popliteal artery on ultrasound, the needle is advanced using an in-plane technique from medial to lateral toward the targeted interspace [35]. Once the needle tip is appropriately positioned, approximately 15–20 mL of local anesthetic is injected. The injectate spreads medially and laterally along the tissue plane between the popliteal artery and the posterior capsule, thereby anesthetizing the terminal articular branches supplying the posterior knee while minimizing involvement of the major motor nerve trunks [15,16,20,52].

#### 4.2.2. The PPB Approach

The target of the PPB is located slightly more proximal or lateral than that of the IPACK block. Specifically, the PPB targets the dense neural plexus surrounding the adductor hiatus, where the superficial femoral artery transitions into the popliteal artery, rather than the posterior capsule itself [34]. To perform the block, the ultrasound transducer is used to trace the superficial femoral artery distally along the sartorius muscle. As the artery courses through the adductor hiatus and continues as the popliteal artery, the needle is advanced in-plane, typically from lateral to medial, toward the space between the femoral shaft and the posterior wall of the popliteal artery [36]. Depending on patient anatomy, ultrasound visualization, and operator preference, the needle may be advanced using either a lateral-to-medial or medial-to-lateral in-plane approach. Regardless of the trajectory, the target is the fascial plane adjacent to the popliteal artery, and the needle tip is commonly positioned at approximately the 1–2 o’clock or 10 o’clock position relative to the artery, depending on the chosen approach and sonographic window. Once the needle is appropriately positioned, local anesthetic is injected into the perivascular space surrounding the arterial adventitia. Unlike the IPACK technique, which aims to deposit injectate between the femoral shaft and the posterior capsule, the PPB relies on perivascular spread around the popliteal artery. This allows the local anesthetic to distribute throughout the popliteal fossa and envelop the popliteal plexus more comprehensively. Cadaveric dye studies have demonstrated that this injection pattern consistently stains the posterior articular branches supplying the knee joint, supporting the anatomical rationale and effectiveness of the PPB for posterior knee analgesia [15,17].

### 4.3. Procedural Difficulty, Ergonomics, and Substitutability

**Figure 3:**
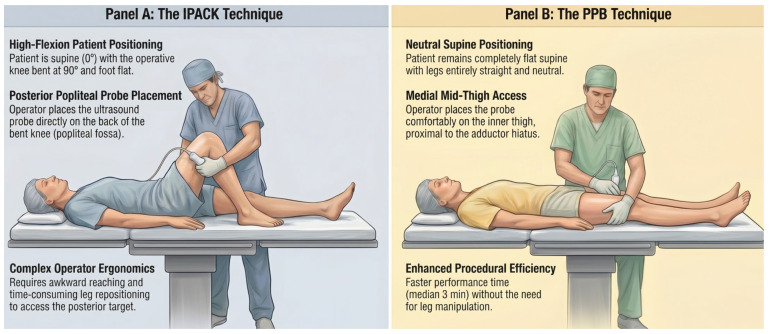
Ergonomics and positioning in IPACK vs. PPB Illustration created using Gemini (3.5 Flash) based on the procedural positioning described by Fujino et al. (2025) [17] and refined by the authors to reflect their interpretation of the technique.

Ergonomic efficiency and procedural time are important considerations for the clinical adoption of a regional anesthesia technique in a fast-paced operating room (OR) environment [37]. Available clinical studies suggest that the PPB may offer ergonomic advantages over the IPACK block, including shorter procedural time and the ability to be performed without patient repositioning [17,45,51]. The distinct procedural setups and operational workflows for both techniques are visually contrasted in Figure 3 [17,37].

**Figure 4:**
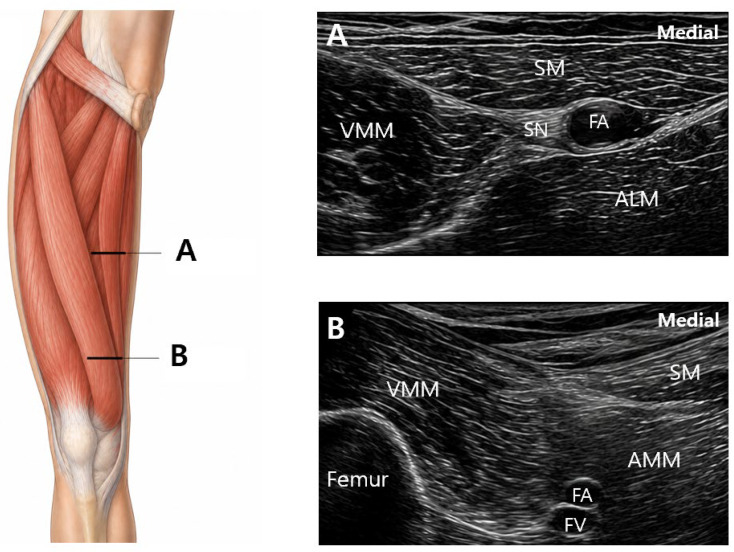
Comparative ultrasound anatomy illustrating the target sites for proximal ACB (**A**) and distal PPB (**B**). The anatomical illustration was created using ChatGPT (GPT-5.5 ) and reviewed by the authors. The ultrasound images are original clinical images acquired by the authors. Abbreviations: ACB, Adductor canal block; ALM, Adductor longus muscle; AMM, Adductor magnus muscle; FA, Femoral artery; FV, Femoral vein; PPB, popliteal plexus block; SM, Sartorius muscle; VMM, Vastus medialis muscle.

As shown in Figure 4, the sonographic transition from the mid-thigh (A) to the adductor hiatus (B) delineates the anatomical shift from isolated sensory nerve (saphenous) coverage to the complex popliteal plexus formation [31]. The movement from A to B represents the clinical transition from ACB (targeting the saphenous nerve) to the PPB (targeting the perivascular plexus near the adductor hiatus), effectively bridging the posterior analgesic gap without requiring the demanding patient repositioning mandated by traditional IPACK approaches [17,31,34].

#### 4.3.1. Ergonomics and Patient Positioning

One practical limitation of the IPACK block is that, depending on the technique used, the ultrasound probe is positioned either over the distal medial thigh or the popliteal fossa. In many cases, optimal probe placement requires some degree of lower-limb repositioning, such as external rotation or a frog-leg position, to improve access and sonographic visualization [13,46]. In some patients, particularly those with advanced age, obesity, or severe knee deformity or contracture, repositioning may be uncomfortable and can reduce the quality of the sonographic window because of altered anatomy or prominent femoral condyles [46]. In contrast, the PPB can be performed with the patient in the neutral supine position without additional repositioning, allowing the operator to advance the transducer distally from the preceding ACB or FTB site [17,51]. This workflow may offer ergonomic advantages and improve procedural convenience; however, these potential benefits have been reported in a limited number of clinical studies and require further validation.

#### 4.3.2. Performance Time

One randomized controlled trial reported a shorter median procedural time for the PPB (3 min) than for the IPACK block (5 min; *p* < 0.0001) [17]. Although this finding may reflect potential ergonomic advantages of the PPB, its clinical and economic significance has not yet been established and requires further investigation.

#### 4.3.3. Substitutability

Current evidence suggests that the PPB may represent a promising alternative to the IPACK block for posterior knee analgesia after total-knee arthroplasty. Available randomized clinical trials indicate that the PPB provides analgesia comparable to IPACK while preserving motor function, with additional practical advantages including shorter procedural time, decreased postoperative nausea, and elimination of patient repositioning in some clinical settings [15,17,41]. However, these findings are primarily derived from a limited number of single-center randomized trials with relatively short follow-up periods and should therefore be interpreted with appropriate caution.

Although a shorter procedural time may improve workflow efficiency, its clinical and economic significance remains uncertain and is likely to vary according to institutional practice and surgical volume [53]. Similarly, while no major safety concerns have been reported in the currently available clinical studies, the evidence remains limited. One case of inadvertent arterial puncture without long-term sequelae has been described, highlighting the importance of meticulous ultrasound guidance and careful needle placement during the procedure [17].

Overall, the PPB appears to be a feasible and anatomically well-founded motor-sparing technique that may serve as an effective alternative to IPACK. Nevertheless, larger multicenter randomized trials with longer follow-up are needed before the PPB can be recommended as a routine replacement for IPACK.

To facilitate interpretation of the available evidence, Table 2 provides a concise summary of the key anatomical and clinical studies on the PPB. The table includes the study design, sample size, primary outcome, assessment timepoint, and principal findings, thereby allowing readers to appreciate the relative strength of the available evidence.

## 5. Clinical Limitations and Technical Challenges of PPB

While the PPB may offer ergonomic advantages while providing analgesia comparable to that of the IPACK block [15,17], its clinical application also presents specific anatomical and technical limitations that warrant careful consideration.

### 5.1. Vascular Proximity and Arterial Puncture Risk

Anatomically, the PPB targets the adventitia of the popliteal artery at the adductor hiatus, where the superficial femoral artery transitions into the popliteal artery. Because the injection is performed in a highly perivascular region, the needle trajectory lies in close proximity to major vascular structures [15,17]. Although current clinical studies have reported no serious complications, such as persistent motor deficits or falls, one case of inadvertent arterial puncture without long-term sequelae has been described [17]. Consequently, meticulous ultrasound-guided visualization of vascular structures, incremental injection with frequent aspiration, and careful needle control are essential to minimize the risk of inadvertent intravascular injection, local anesthetic systemic toxicity (LAST), and hematoma formation [17,45,54].

Particular caution should be exercised in patients receiving anticoagulant therapy, those with peripheral arterial disease, frail elderly patients, morbid obesity, or distorted regional anatomy, in whom vascular identification and needle placement may be technically more challenging. Postoperative neurovascular assessment is also advisable to facilitate early recognition of rare vascular or neurological complications.

Although the use of lower injectate volumes has been proposed as a potential strategy to reduce vascular spread and the risk of LAST, the optimal local anesthetic volume for the PPB has not yet been established. A recently published randomized controlled trial protocol has been designed to compare different injectate volumes and may help define the optimal dosing strategy in future clinical practice [55].

### 5.2. Technical Dependency and Sonographic Learning Curve

Although the PPB may offer procedural advantages, including a shorter procedural time and the ability to be performed in the neutral supine position without patient repositioning [17], the technique still requires a high level of sonographic expertise. The operator must accurately trace the superficial femoral artery as it dives deep into the adductor hiatus and safely advance the needle to reach the narrow perivascular space [15,17]. For novice anesthesiologists, identifying this deep transition zone can be technically challenging, especially in morbidly obese patients or those with severe anatomical deformities, potentially leading to inconsistent block success rates compared with simpler superficial plane blocks [17]. The clinical efficacy of the PPB depends on adequate interfascial spread of the local anesthetic. Cadaveric studies have demonstrated that injectate spread to the popliteal fossa is influenced by the fascial anatomy of the distal adductor canal, including the vastoadductor membrane. Inadequate distal spread beyond this fascial barrier may contribute to incomplete coverage of the popliteal plexus, potentially contributing to technical variability and the sonographic learning curve associated with the procedure [18].

### 5.3. Need for Comprehensive Long-Term Data

Finally, as a relatively novel regional anesthesia technique, the current literature on the PPB is still dominated by cadaveric studies and early-phase randomized controlled trials [15,17,41]. In addition, the available clinical studies demonstrate considerable methodological heterogeneity, including differences in local anesthetic type, concentration, and volume, as well as variations in perioperative analgesic protocols and institutional surgical pathways. Consequently, although early non-inferiority trials suggest that the PPB may be a promising motor-sparing alternative to the IPACK block, the current evidence remains limited [17]. Further large-scale, multicenter prospective studies with standardized procedural protocols are needed to better define optimal practice, evaluate reproducibility, and determine the role of the PPB in more complex clinical settings, such as revision TKA.

### 5.4. Current Clinical Indications and Evidence Gaps

Current evidence supporting the PPB is derived predominantly from patients undergoing primary unilateral TKA within standardized multimodal analgesia and ERAS protocols [14,15,17]. However, the available evidence remains limited by relatively small sample sizes, the predominance of single-center studies, heterogeneous block techniques and perioperative analgesic protocols, and potential operator dependence. Although the PPB has demonstrated favorable early clinical outcomes in experienced hands, successful implementation requires familiarity with the perivascular sonoanatomy of the adductor hiatus and proficiency in ultrasound-guided regional anesthesia. Consequently, a learning curve is likely to exist, particularly for clinicians with limited experience in deep perivascular nerve blocks.

In addition, the reproducibility of the PPB in non-specialized clinical settings has not yet been fully established. Most published studies have been conducted at specialized centers by experienced regional anesthesiologists, and it remains uncertain whether similar procedural success, motor-sparing characteristics, and analgesic outcomes can be consistently achieved in routine clinical practice or lower-volume institutions. Standardized procedural protocols, structured training, and future multicenter studies involving operators with varying levels of experience will therefore be important to evaluate the generalizability of this technique.

Therefore, the current findings should not be generalized to revision TKA, bilateral procedures, patients with severe deformity or contracture, outpatient arthroplasty, individuals with significant peripheral vascular disease, or those receiving anticoagulant therapy, as evidence in these clinical settings remains limited or unavailable. Further multicenter randomized studies using standardized protocols are required before broader clinical recommendations can be made.

Until such evidence becomes available, the PPB should be considered a promising motor-sparing technique whose broader implementation should be guided by institutional expertise, operator experience, and appropriate patient selection.

## 6. Conclusions and Future Directions

TKA is associated with substantial postoperative pain, and effective analgesia is a key determinant of functional recovery and patient satisfaction. Traditional regional anesthesia techniques targeting major nerve trunks, such as the femoral or sciatic nerve, often provide effective analgesia at the expense of motor impairment. Consequently, contemporary regional anesthesia has increasingly shifted toward selective motor-sparing approaches that preferentially target the sensory innervation of the osteotomes and joint capsule while preserving motor function.

Within this evolving paradigm, the combination of the ACB and PPB represents a rational anatomical strategy for comprehensive anterior and posterior knee analgesia. ACB primarily addresses anterior knee pain, whereas the PPB targets the terminal articular branches surrounding the adductor hiatus and popliteal artery, providing posterior capsular analgesia while preserving distal motor function.

The currently available randomized clinical trials suggest that the PPB provides analgesia comparable to the IPACK block while maintaining motor preservation. In addition, individual studies have reported practical advantages, including shorter procedural time and reduced postoperative opioid consumption and postoperative nausea. However, these findings are derived from a limited number of predominantly single-center randomized trials with relatively short follow-up periods and should therefore be interpreted with appropriate caution.

Taken together, the available anatomical and clinical evidence suggests that the PPB is a promising, anatomically sound, and motor-sparing alternative to the IPACK block within multimodal analgesic strategies for TKA. Although its ergonomic advantages and encouraging early clinical outcomes support further adoption and investigation, additional multicenter randomized studies with standardized protocols and longer follow-ups are required before the PPB can be recommended as a routine replacement for IPACK in contemporary clinical practice.

## Figures and Tables

**Table 1:** Evolution of the Regional Anesthesia Paradigm in TKA.

Era (Paradigm)	Primary Technique	Anatomical Target	Motor Preservation	Clinical Limitations
Traditional (Trunk Block)	FNB + Proximal SNB	Main nerve trunks (Dermatomal/Myotomal)	Poor (Complete paralysis of quadriceps & lower leg)	High risk of falls, precludes early ERAS ambulation [11].
Transitional (Anterior only)	ACB alone	Saphenous N. & NVM (in Adductor Canal)	Excellent (Anterior only)	Fails to control severe posterior knee pain (Blind spot) [14,15].
Modern (Anterior + Posterior)	ACB + IPACK	Anterior canal + Posterior interspace (Capsular)	Excellent	Ergonomically difficult (requires repositioning), longer procedural time [16,17].
Advanced (Targeted Plexus)	ACB + PPB	Anterior canal + Popliteal Plexus (Perivascular)	Excellent	Requires familiarity with perivascular sonoanatomy, close proximity to the popliteal artery [18], limited long-term clinical evidence.

Abbreviations: ACB, adductor canal block; FNB, Femoral nerve block; SNB, Sciatic nerve block; IPACK, infiltration between the popliteal artery and the capsule of the knee; PPB, Popliteal plexus block; ERAS, Enhanced recovery after surgery, NVM, nerves to the vastus medialis.

**Table 2:** Summary of key anatomical and clinical evidence supporting popliteal plexus block and IPACK block.

Study	Design	Population (n)	Block Combination	Local Anesthetic Regimen	Primary Outcome (Time Point)	Main Findings
Tran et al., 2019 [22]	Cadaveric	15 cadaveric specimens	Anatomical dissection	Not applicable	Posterior capsule innervation	Defined popliteal plexus anatomy
Runge et al., 2018 [34]	Feasibility	10 patients undergoing primary unilateral TKA	FTB + PPB	FTB: 10 mL 0.5% bupivacaine with epinephrine (1:200,000); PPB: 10 mL 0.5% bupivacaine with epinephrine (1:200,000)	Pain score (1 h)	Significant pain reduction without motor block
Sørensen et al., 2025 [41]	Single-center RCT	165 patients undergoing primary unilateral TKA	PPB + FTB vs. FTB vs. ACB	PPB: 10 mL 0.5% bupivacaine; FTB: 15 mL 0.5% bupivacaine; ACB: 25 mL 0.5% bupivacaine	Opioid consumption (24 h)	Reduced opioid consumption; no difference in pain or mobilization Oxycodone consumption −4 mg (95% CI −7.4 to −1.0) compared with FTB; −6 mg (95% CI −8.3 to −1.3) compared with ACB
Fujino et al., 2025 [17]	Non-inferiority RCT	86 patients undergoing elective primary TKA	PPB + FTB vs. IPACK + FTB	PPB: 15 mL 0.25% levobupivacaine; IPACK: 15 mL 0.25% levobupivacaine; FTB: 10 mL 0.25% levobupivacaine	Posterior pain (8 h)	Non-inferior analgesia; shorter procedure time 3 vs. 5 min; lower PONV Difference 4.7% (90% CI −5.6 to 14.9); non-inferiority achieved
Stebler et al., 2026 [15]	Double-blind RCT	66 patients undergoing elective TKA	CFNB + PPB vs. CFNB	PPB: 15 mL 0.5% ropivacaine; CFNB: continuous 0.2% ropivacaine infusion	Morphine-equivalent consumption (12 h)	Lower opioid use (6.1 vs. 10.0 mg); benefit not sustained at 24 h

The quantitative values presented in this table are derived from individual published studies and are provided to illustrate representative findings. They should not be interpreted as pooled estimates from a formal meta-analysis. Abbreviations: ACB, Adductor canal block; CFNB, continuous femoral nerve block; FTB, Femoral triangle block; IPACK, infiltration between the popliteal artery and the capsule of the posterior knee; PONV, postoperative nausea and vomiting; PPB, popliteal plexus block.

## Data Availability

No new data were created or analyzed in this study. Data sharing is not applicable to this article.
